# Empowerment of solitude: interactive impact of positive solitude, self-esteem, and hope on college students' life orientation

**DOI:** 10.3389/fpsyg.2026.1792649

**Published:** 2026-04-15

**Authors:** Ze Peng, Zhihua Li

**Affiliations:** 1 School of Education, Hunan University of Science and Technology, Xiangtan, China; 2 Mental Health Education Center, Xiangtan Institute of Technology, Xiangtan, China

**Keywords:** college students, hope, life orientation, positive solitude, self-esteem

## Abstract

**Objective:**

This study aimed to examine the association between positive solitude (PS) and life orientation among Chinese college students, and the mediating role of self-esteem and the moderating role of hope in this relationship.

**Method:**

A cross-sectional survey was conducted among 830 Chinese undergraduate and graduate students. The validated instruments—including the Positive Solitude Behavior Scale (PSBS), the Life Orientation Test–Revised (LOT-R), the Adult Trait Hope Scale (ATHS), and the Rosenberg Self-Esteem Scale (RSES)—were used to assess the constructs of interest. Gender was included as a covariate in all analytical models.

**Results:**

(1) After controlling for gender, positive solitude exhibited a significant positive direct effect on life orientation, (2) self-esteem significantly mediated the relationship between positive solitude and life orientation, and (3) hope moderated the first stage of this mediation pathway—specifically, higher levels of hope strengthened the positive association between positive solitude and self-esteem, thereby amplifying the indirect effect of positive solitude on life orientation through self-esteem.

**Conclusion:**

Positive solitude contributes to enhanced life orientation among college students both directly and indirectly by bolstering self-esteem. Moreover, dispositional hope is a salient psychological resource that intensifies this indirect pathway, underscoring its protective and facilitative function in promoting adaptive developmental outcomes.

## 1 Introduction

In recent years, the attitudes of Chinese college students toward their future lives have exhibited a distinct “suspended” characteristic, marked by a coexistence of confusion and tension arising from diverse explorations (Feng et al., 2022). This phenomenon stems from the confluence of social transformation, economic shifts, technological revolutions, and the awakening of consciousness among contemporary college students (Barth et al., 2007). The college period represents a critical stage for personality development and the transition to socialization; when facing the future, a positive mindset facilitates the development of clearer goals (Liang, 2023). As a core construct in positive psychology, optimism has been empirically validated as a key predictor of individuals' physical and mental health, subjective wellbeing, and social adaptability (Gaudreau and Blondin, 2004; Bortolotti, 2018). In psychological research, “Life Orientation” (LO) is frequently used to denote an individual's general dispositional tendency toward future expectations, with its core being dispositional optimism—a construct that shapes how individuals cope with stress, set goals, and navigate challenges (Carver et al., 2010; Oleś et al., 2013). Life orientation is not a unidimensional construct; instead, it is a continuum comprising two relatively independent dimensions: optimism and pessimism (Hinz et al., 2022). Life orientation exerts profound influences on individuals' adversity resilience and achievement motivation, among other factors (Russo-Netzer and Tarrasch, 2024; Stavrulaki et al., 2021). The life orientation of college students is shaped by multiple factors, including personal traits and social environments (Jovanović and Gavrilov-Jerković, 2013). Notably, a “transitional orientation”—characterized by active engagement with developmental tasks—is associated with more optimistic traits (Paszkowska-Rogacz, 2024). Thus, exploring the factors that influence college students' life orientation holds significant theoretical and practical value.

According to the self-determination theory (SDT), solitary behavior can be categorized as self-determined and non-self-determined types (Nicol, 2005). Building on this framework, Chinese scholar Chen et al. (2012) further subdivided self-determined solitude into “positive solitude (PS)”—accompanied by positive emotional experiences—and “solitary withdrawal”—marked by complex, often negative, emotional states. Positive solitude (PS) refers to a behavioral orientation in which individuals actively choose to be alone and derive positive emotional experiences from the act; it represents a prototypical means of satisfying the autonomy need as posited by SDT (Deci and Ryan, 2000). When college students proactively choose solitude to fulfill higher-order psychological needs, they gain a sense of control over their time and space—a perception that strengthens their confidence in their own abilities and may thereby foster positive future expectations (Dai et al., 2011). PS provides a cognitive restructuring space for life orientation, enabling individuals to challenge negative thought patterns and develop a more positive explanatory style (Cayubit, 2024). Additionally, PS facilitates the exploration and integration of life meaning, and a clear sense of life meaning serves as a solid foundation for an optimistic life orientation (Russo-Netzer and Tarrasch, 2024). Moreover, PS functions as a strategy for restoring and replenishing psychological resources; when individuals possess sufficient resources, they are more likely to approach the future with an optimistic mindset (Hobfoll, 1989). Investing time in PS for long-term self-improvement activities enhances self-efficacy, which in turn strengthens positive future expectations (Busseri, 2013). However, empirical research directly investigating the mechanism by which PS influences college students' life orientation remains limited.

**Hypothesis 1:** Positive solitude (PS) has a direct positive effect on the life orientation of college students.

Self-esteem is defined as an individual's global evaluation of their own worth and capabilities (Rosenberg, 1965) and serves as a critical mediating mechanism linking behavior to psychological adaptation. SDT suggests that PS promotes self-exploration and growth by satisfying the needs for autonomy and competence, thereby enhancing self-esteem (Friedrich and Schütz, 2025). The cognitive consistency theory posits that the self-growth experiences derived from PS align with an individual's self-value cognition, reinforcing high self-esteem (Markowski and Serpe, 2021). Individuals with high self-esteem, due to their firm belief in their abilities, tend to interpret future events through a positive lens, thus forming a more optimistic life orientation (Duman and Wang, 2025). Within the psychological capital framework, positive solitude—an active form of self-investment—facilitates the accumulation of psychological capital (including self-esteem); both concentration and reflection during solitude enhance individuals' sense of competence and self-worth (Thomas and Azmitia, 2019), which in turn strengthens their confidence and optimism toward the future (Caprara et al., 2025). Long-term engagement in positive solitude has been shown to significantly enhance self-esteem levels (Borg and Willoughby, 2022, 2023). Previous studies have indicated that self-esteem may mediate the relationship between positive solitude and life orientation (Jackman and MacPhee, 2017; Verdugo et al., 2018). Longitudinal research has further demonstrated that controlling for self-esteem significantly attenuates the direct association between positive solitude and life orientation (Ma, 2025). Thus, positive solitude may enhance life orientation by elevating self-esteem.

**Hypothesis 2:** Self-esteem mediates the relationship between positive solitude and the life orientation of college students.

Hope is a goal-based positive psychological state consisting of two core components: pathway thinking (the ability to generate strategies to achieve goals) and agency thinking (the motivation to pursue those strategies) (Snyder, 1994). Research has shown that hope training enables college students to use solitude more effectively to enhance self-awareness and self-esteem (Kirby et al., 2021). Conversely, individuals with low hope may experience “aimless emptiness” during solitude due to a lack of goal orientation, making it difficult to derive value confirmation from the experience and thus weakening the positive effect of solitude on self-esteem (Munoz et al., 2020). As a bridge connecting the “present self” and the “future self,” hope transforms the “I am capable” belief inherent in high self-esteem into concrete action plans through pathway and agency thinking, thereby reinforcing positive future expectations more effectively (Hu et al., 2022). Similarly, in the context of PS, college students with high hope can more effectively translate the self-esteem gained from solitude into long-term positive orientations toward their academic and personal lives. In contrast, individuals with low hope may struggle to convert high self-esteem into clear, optimistic future expectations due to deficits in pathway-planning abilities (Uzondu and Hollenbaugh, 2025). The moderating effect of hope is subject to boundary conditions, including the quality of solitude and the individual's developmental stage (Bryce et al., 2021). At its core, this effect reflects a “resource activation” process: high hope activates the growth potential inherent in PS and mitigates the anxiety associated with the uncertainty of solitude (Ojala, 2023).

**Hypothesis 3:** Hope moderates the mediated relationship between positive solitude and life orientation through self-esteem.

## 2 Materials and methods

### 2.1 Participants

A random sampling method was used to select 830 college students from three universities in Hunan Province as participants. The participants were guided by trained peer college student cadres, and consent was obtained from the participants to answer questions online, and the data were exported from the background of the Golden Data System after completion. The participants included 300 male college students (36.1%) and 530 female college students (63.9%), aged between 16 and 24 years (*M* = 19.79; SD = 1.14).

### 2.2 Instruments

#### 2.2.1 Positive solitude behavior scale (SBS)

The positive solitude behavior scale developed by Chen et al. (2012) mainly reflects positive psychological traits of individuals. It consists of 10 items and is scored on a 5-point Likert scale, with 1 representing “strongly disagree” and 5 representing “strongly agree.” The total score is calculated by adding up the items, and the higher the total score, the higher the component of PS behavior. The Cronbach's α coefficient of the scale in this study was 0.948.

#### 2.2.2 Life orientation test (LOT)

The life orientation test (LOT), developed by Scheier et al. (1994), was utilized to assess individuals' general tendency toward positive expectations regarding future outcomes. This scale uses a 5-point Likert-type response format (ranging from “strongly disagree” to “strongly agree”), comprising three positively worded items (e.g., “In uncertain situations, I usually expect the best”) and three negatively worded items (e.g., “For me, if something can go wrong, it will”). Responses are scored on a 0–4 scale (with “strongly disagree” = 0 and “strongly agree” = 4), and negatively worded items are reverse-coded. Higher total scores indicate greater dispositional optimism. The Cronbach's α coefficient for the LOT in this study was 0.634.

#### 2.2.3 Adult Trait Hope Scale (THS)

The Adult Trait Hope Scale, developed by Snyder (2002), mainly measures hope levels in individuals aged 15 and above. The scale covers two aspects of path thinking and dynamic thinking, with 12 items. Questions 1, 4, 6, and 8 are used to measure path thinking; questions 2, 9, 10, and 12 are used to measure dynamic thinking; and questions 3, 5, 7, and 11 are used to divert the subjects' attention, not for scoring. The 4-point Likert scoring system is used, and the total score is calculated by adding up each item. The higher the total score, the higher the individual's level of hope. The scale is widely used in China and has good reliability and validity. The Cronbach's α coefficient of the scale in this study was 0.881.

#### 2.2.4 Self-esteem scale (SES)

The self-esteem scale developed by Rosenberg was used and revised by Chinese scholars (Wang et al., 1997). The 10 questions were scored on a 4-level scale, with “1” representing very inconsistent and “4” representing very consistent. Among them, questions 1, 2, 4, 6, and 7 were positively scored, and the rest were negatively scored, with higher scores indicating higher self-esteem levels for the individual. The Cronbach's α of the scale was 0.830 in this study.

### 2.3 Data analysis

Data analysis was performed using SPSS 26.0 (International Business Machines Corporation (IBM), United States of America) with the PROCESS macro program in this study. The former was used to perform common method bias tests, descriptive statistics, and correlation analyses. The latter used Model 7 in the process macro program using the Bootstrap method to test the mediating effect and moderated mediating effects in the data, with a set of 5,000 extractions and a confidence interval of 95% to ensure more robust results.

### 2.4 Common method bias test

In this study, the Harman's single-factor test was used to assess common method variance. The results indicated that six factors had eigenvalues greater than 1. After conducting an unrotated exploratory factor analysis, the first factor accounted for 27.445% of the variance, which was below the critical value of 40% (Xiong et al., 2012). This suggests that no significant common method variance was detected in this study.

## 3 Results

### 3.1 The correlations among various psychological variables

This study aims to explore the relationships among PS, life orientation, hope, and self-esteem. Descriptive statistics and correlation analysis results for the key variables are presented in Table 1. For Chinese college students, PS, life orientation, and self-esteem were all at above-average levels, while hope was at an average level. Notably, PS exhibited the greatest individual variability (*SD* = 9.459), which provides a necessary prerequisite for subsequent analyses. Correlation analysis revealed that positive solitude behavior was significantly and positively associated with life orientation, hope, and self-esteem, respectively (*p* < 0.01). Specifically, the correlation between positive solitude behavior and hope was the strongest (*r* = 0.441), whereas the correlation with self-esteem was the weakest (*r* = 0.296).

**Table 1:** Descriptive statistics and correlation analysis results.

Variables	*M*	SD	Positive solitude	Life orientation	Hope	Self-esteem
Positive solitude	33.765	9.459	1			
Life orientation	21.561	3.376	0.344^**^	1		
Hope	20.638	4.557	0.441^**^	0.390^**^	1	
Self-esteem	27.377	4.448	0.296^**^	0.358^**^	0.353^**^	1

^**^p < 0.01.

### 3.2 The mediating role of self-esteem in the relationship between positive solitude and life orientation

To examine the mediating role of self-esteem in the relationship between positive solitude and life orientation, this study utilized Model 4 of the PROCESS macro (Mittal, 2019) with gender as a control variable. The sample comprised 830 participants, and the significance of the mediating effect was assessed via the bias-corrected Bootstrap method (5,000 resamples). A regression analysis with self-esteem as the dependent variable revealed that PS significantly and positively predicted self-esteem (β = 0.137, *t* = 8.073, *p* < 0.001). The model was statistically significant, *F*_(2, 827)_ = 36.676, *p* < 0.001, accounting for 8.8% of the variance in self-esteem (*R*^2^ = 0.088). For the regression model with life orientation as the dependent variable (including both positive solitude and self-esteem as predictors), both self-esteem (β = 0.210, *t* = 7.243, *p* < 0.001) and positive solitude (β = 0.085, *t* = 6.629, *p* < 0.001) emerged as significant positive predictors. This model exhibited a good fit, *F*_(3, 826)_ = 67.449, *p* < 0.001, explaining 20.8% of the variance in life orientation (*R*^2^ = 0.208). In the total effect model, positive solitude exerted a significant total effect on life orientation (*c*' = 0.113, *t* = 9.105, *p* < 0.001). Mediating effect analysis indicated that after incorporating self-esteem as a mediator, the direct effect of positive solitude on life orientation remained significant (*c*' = 0.085, *t* = 6.629, *p* < 0.001). The indirect effect of positive solitude on life orientation through self-esteem was 0.029, with a bootstrap 95% confidence interval of (0.019, 0.039) (excluding zero), confirming its significance. The effect size analysis showed that the indirect effect accounted for 25.3% of the total effect (*ab/c*' = 0.253) and 33.9% of the direct effect (*ab/c*' = 0.339). The fully standardized indirect effect was 0.081. In summary, self-esteem serves as a partial mediator between positive solitude and life orientation, where positive solitude not only directly enhances individuals' life orientation but also indirectly fosters it by promoting self-esteem.

### 3.3 The moderating role of hope

**Figure 1:**
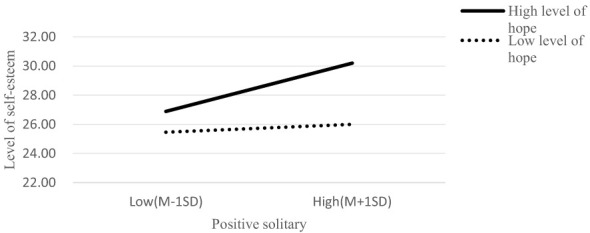
Moderating effect of college students' hope between positive solitude and self-esteem.

Subsequently, Model 7 of the PROCESS macro was used to test the moderated mediation effect. Controlling for gender, results (see Table 2) revealed that the interaction term between positive solitude and hope significantly predicted college students' self-esteem in a positive direction (β = 0.016, *t* = 4.712, *p* < 0.001). This indicates that hope moderates the indirect pathway through which positive solitude influences life orientation via self-esteem. To further probe the interaction effect, college students were grouped into high- and low-hope subgroups using the mean ± 1 standard deviation (*M* ± 1*SD*) criterion, followed by a simple slope analysis (see Figure 1). Results showed that when hope levels were low (*M* – 1*SD*), the positive predictive effect of positive solitude on self-esteem was non-significant [β_simple_ = 0.029, *t* = 1.53, *p* > 0.05, 95% CI = (−0.005, 0.071)]. In contrast, when hope levels were high (*M* + 1*SD*), this positive predictive effect was significantly strengthened [β_simple_ = 0.175, *t* = 4.86, *p* < 0.01, 95% CI = (0.022, 0.052)]. These findings suggest that the positive association between positive solitude and self-esteem is amplified with increasing levels of hope. The bootstrap test results further confirmed that when hope scores exceeded the sample mean, the 95% confidence interval for the indirect effect did not include zero, indicating a significant positive predictive effect of positive solitude on self-esteem. Moreover, higher hope levels were associated with a stronger indirect effect of positive solitude on life orientation through self-esteem.

**Table 2:** Moderated mediating effect tests.

Variable	Self-esteem		Life orientation	
	***Beta***.	***t***	***Beta***.	***t***
Gender	−0.136	−0.451	−0.963	−4.333^**^
Positive solitude	0.102	4.912^**^	0.085	6.629^**^
Self-esteem			0.210	7.243^**^
Hope	0.308	6.434^**^		
Positive solitude × hope	0.016	4.712^**^		
*R^2^*	0.186		0.208	
*F*	32.608^**^		67.449^**^	

^**^p < 0.01.

## 4 Discussion

This study aims to explore the complex relationship among positive solitude, self-esteem, hope, and life orientation among college students. Based on questionnaire survey data from 830 Chinese college students and using a moderated mediation model for analysis, all three core hypotheses of this study were supported by the empirical results. First, after controlling for the gender variable, positive solitude can directly and significantly predict college students' life orientation positively; thus, Hypothesis 1 is established. Second, self-esteem plays a significant partial mediating role between positive solitude and life orientation, meaning that positive solitude not only directly affects life orientation but also indirectly enhances it by improving self-esteem, thus Hypothesis 2 is established. Finally, hope moderates the first half of this mediation model; that is, the higher the level of hope, the stronger the indirect effect of positive solitude on life orientation through self-esteem, thus Hypothesis 3 is also established. These findings collectively reveal an internal mechanism: when college students actively choose and can gain positive experiences in solitude, their self-esteem levels will increase, and they will hold more optimistic expectations for the future (life orientation). The effectiveness of this transformation process is largely determined by the individual's hope for achieving goals.

### 4.1 The direct impact of positive solitude on college students' life orientation

The results of this study found that positive solitude significantly and positively predicted college students' life orientation, which is highly consistent with the core view of self-determination theory (Li et al., 2026). According to the self-determination theory, autonomy is one of the basic psychological needs of human beings. When individuals actively choose a certain behavior out of intrinsic motivation, their psychological needs are met, thereby promoting personal growth and integration. Positive solitude is a typical self-determined behavior, which gives individuals a sense of control over time and space, satisfying their autonomy needs. This sense of control and autonomy is an important foundation for forming positive self-perception and future expectations. In this study, the significant positive correlation between positive solitude and life orientation (*r* = 0.344, *p* < 0.01) and the stable predictive effect in the regression analysis (β = 0.085, *t* = 6.629, *p* < 0.001) support the hypothesis that “behavior can shape personality traits.”

From the perspective of the conservation of resources theory, positive solitude is an important strategy for restoring and investing psychological resources (Sheeja, 2026). In today's high-pressure, fast-paced social environment, college students often face multiple challenges such as academic, interpersonal, and future planning demands, and their psychological resources are easily depleted. Positive solitude provides a valuable “psychological downtime,” allowing individuals to temporarily withdraw from external stimuli and social comparisons, engage in self-reflection, emotional regulation, and energy replenishment. The retention and acquisition of resources are key to coping with stress (Ma, 2025). When college students restore their psychological resources through positive solitude (such as reading, meditation, and developing personal interests), they are more capable and confident of facing the uncertainties of the future with an optimistic and proactive attitude, setting more challenging goals, and thus forming a more positive life orientation.

In addition, positive solitude provides a space for cognitive restructuring (Cayubit, 2024). During solitude, individuals have the opportunity to deeply reflect on past experiences and future possibilities, challenge existing negative thinking patterns, and establish more positive and adaptive explanatory styles (Krok and Telka, 2019). For example, a student who has been frustrated in social interactions may reinterpret the experience as a learning opportunity rather than a self-denial when alone, and this cognitive transformation helps to form an optimistic expectation that “even when encountering difficulties, the future can still be positive.” The results of this study suggest that encouraging college students to develop high-quality, purposeful solitude habits may be an effective way to alleviate the widespread sense of future “suspension” and confusion among contemporary youth and enhance their psychological resilience and sense of purpose.

### 4.2 The mediating role of self-esteem

This study further found that self-esteem plays a partial mediating role between positive solitude and life orientation [*ab* = 0.029, *SE* = 0.005, 95% CI = (0.019, 0.039)], which deepens our understanding of how positive solitude influences life orientation. According to SDT, positive solitude effectively meets the “competence” need by satisfying the need for autonomy and through self-exploration, skill enhancement, and achievement during solitude (Strempfl et al., 2021). When college students successfully complete a task, deepen their understanding, or achieve a self-set goal during solitude, they experience a strong sense of competence and efficacy. This “I can do it” experience is a core component of self-esteem. Therefore, individuals who frequently engage in positive solitude have their self-esteem levels consolidated and enhanced through repeated positive self-feedback.

According to the cognitive consistency theory (Borg and Willoughby, 2022), individuals with high self-esteem tend to view themselves and the world in a more positive and favorable light. They have a firmer belief in their own value, and this belief generalizes to their expectations for the future. An individual who believes they have the ability to handle challenges and deserve a bright future is more likely to form an optimistic life orientation (Friedrich and Schütz, 2025). The regression analysis of this study showed that after introducing the self-esteem variable, the direct effect of positive solitude on life orientation remains significant, but the indirect path (positive solitude → self-esteem → life orientation) was also significant and accounted for 25.3% of the total effect. This clearly indicates that self-esteem is an important transformation mechanism through which positive solitude exerts its long-term psychological benefits. This study not only explains “why” positive solitude makes people more optimistic but also points out “through what” internal process this transformation is achieved. This suggests that educators and psychological interveners, in projects aimed at enhancing college students' optimistic mindsets, should not only directly encourage positive behaviors but also focus on exploring and strengthening individuals' sense of self-worth and achievement during solitude experiences, thereby solidifying self-esteem as an important psychological capital.

### 4.3 The moderating role of hope

This study found that hope moderates the first half of the mediation model, that is, the “positive solitude → self-esteem” link. The simple slope analysis further indicates that the enhancing effect of positive solitude on self-esteem is significant only when an individual's hope level is high; in individuals with a lower hope level, this promoting effect is not obvious. This provides a key perspective for understanding the differences in the benefits of positive solitude. According to the Snyder's hope theory (Snyder, 1994), hope consists of two core components: “agency thinking” (the willpower to pursue goals) and “pathways thinking” (planning the ways to achieve goals). High-hope individuals usually have clear goals and a firm belief in achieving them. In the context of positive solitude, college students with high hope do not passively “waste” their solitude time but actively and consciously transform solitude time into a “strategic resource” serving personal goals (Caprara et al., 2025). A hopeful college student is more likely to use their solitude time for systematic review and filling knowledge gaps. This goal-oriented solitude behavior can generate more direct and intense feelings of achievement and competence confirmation, thereby significantly enhancing self-esteem. On the contrary, individuals with low hope may lack clear goals, and their solitude is more likely to result in “aimless idleness” or mindless entertainment. Although this may be voluntary, it is difficult for them to obtain sufficient feedback for self-growth and value confirmation, thus having a limited reinforcing effect on self-esteem. Therefore, hope plays the role of a “resource activator” or “catalyst” here (Kirby et al., 2021). It determines whether the “raw material” of positive solitude can be efficiently “processed” into the “psychological product” of self-esteem. High hope is like a clear blueprint and a powerful engine, ensuring that time alone is effectively invested in self-construction and maximizing its psychological benefits. This finding has significant practical implications: simply advocating “more solitude” may have varying effects and may be ineffective for those with low hope. A more effective strategy might be the dual cultivation of “hope” and “positive solitude.” By providing hope intervention training to college students, especially those who are confused, a bridge to the future can be built, enabling them to more effectively utilize their alone time and transform the potential of solitude into actual self-esteem enhancement and optimism growth.

### 4.4 Research implications, limitations, and future directions

In summary, this study systematically revealed the internal mechanism and boundary conditions of how positive solitude affects college students' life orientation through a moderated mediation model. The theoretical contribution of this study lies in integrating the self-determination theory, the cognitive consistency theory, and the hope theory, confirming that positive solitude, as an active behavior, nurtures optimism through enhancing self-esteem, and this process is effectively regulated by the individual's goal-oriented psychological capital – hope. This provides new empirical evidence for understanding the dynamic relationship between “behavior – psychological capital – personality traits.”

On the practical level, this study offers specific directions for college students' mental health education and personal development guidance. First, universities and student workers should re-examine the value of solitude, dispel the stereotype that “solitude equals loneliness or social disorder,” and promote the benefits of positive solitude through lectures, workshops, and other activities, guiding students to learn how to plan and manage their alone time and use it for reading, creation, skill learning, career planning, and other growth-oriented activities. Second, mental health courses and counseling should pay more attention to self-esteem building, designing activities to help students recognize their strengths during solitude and self-exploration, accumulate successful experiences, and consolidate their sense of self-worth. Third, and most importantly, intervention projects centered on “hope” should be carried out, helping students set clear and feasible short-term and long-term goals and cultivate thinking habits related to planning paths and overcoming obstacles so that their “alone time” can be efficiently utilized, thereby forming a positive cycle of “positive solitude → enhanced self-esteem → goal approach → strengthened hope → more effective solitude.”

This study also has some limitations, pointing the way for future research. First, this study used a cross-sectional design. Although the study revealed associations and possible mechanisms among variables through theoretical construction and data analysis, it could not confirm causal relationships. Future research can adopt longitudinal tracking or experimental intervention methods to more rigorously test causal directions among variables. Second, the samples were all from colleges and universities in Hunan Province. Although they have a certain representativeness, the conclusions should be applied to college students in different regions and cultural backgrounds across the country with caution. Future research can expand the sampling range and conduct cross-cultural comparisons. Third, this study mainly focused on trait hope. Future research can explore how state hope or specific hope intervention measures affect this mechanism. Finally, the “quality” of solitude is difficult to quantify precisely. Future research can combine experience sampling methods to more finely measure specific activities, motivations, and immediate emotional experiences during solitude to distinguish which nature and which context of solitude can produce the most positive benefits. In conclusion, this study reveals that in the growth picture of contemporary college students, positive solitude is not a blank canvas but a fertile ground of the soul that can be carefully cultivated to yield self-esteem, hope, and an optimistic future. The wisdom of educators lies not only in providing this soil but also in helping students learn the goals to sow and the methods to cultivate.

## 5 Summaries and conclusion

By investigating 830 Chinese college students, this study explored the mechanism by which positive solitude behavior affects life orientation and verified the mediating role of self-esteem and the moderating role of hope. The results showed that positive solitude behavior not only directly and positively predicted life orientation in college students but also indirectly enhanced life orientation by raising self-esteem levels, and hope played a reinforcing role in this process. This finding supports self-determination theory and cognitive consistency theory, emphasizing that positive solitude as a self-determined behavior can promote psychological adaptation by satisfying autonomy and basic psychological needs. This study provides empirical evidence for understanding the optimistic tendency cultivation among college students and suggests that educators should encourage positive solitude to enhance psychological capital. In summary, this study reveals the interaction effect of positive solitude, self-esteem, and hope; examines moderate mediating models; reveals the complex mechanism of positive solitude behavior on college students' life orientation; the moderating effect of hope emphasizes the importance of individual differences in psychological intervention; provides a basis for personalized support; and has practical value for college students' mental health interventions. Future studies could be extended to different cultural backgrounds or use longitudinal designs to verify the universality of the model.

## Data Availability

The raw data supporting the conclusions of this article will be made available by the authors, without undue reservation.
